# Supplementary material for: Relationship of parenting styles on depression, anxiety, stress and self-esteem of adolescents
Source: PLoS One. 2025 Dec 3;20(12):e0332854. doi: 10.1371/journal.pone.0332854 (PMC12674523; doi:10.1371/journal.pone.0332854)
Supplement: S1 Table — https://doi.org/10.6084/m9.figshare.30490772 (PDF) [file pone.0332854.s001.pdf]

Study variables

Table A. Study variables of the research

**Variables**	**Definition of variables**	**Measurements**
**A. Dependent Variables**		
Depression	Level of symptoms of depression distinguished by DASS-21 scale.	Normal (0-9), Mild Depression (10-13), Moderate Depression (14-20), Severe Depression (21-27) and Extremely Severe Depression (28+)<br>No Depression (0-9)<br>Depression (> 9)
Anxiety	Level of symptoms of anxiety distinguished by DASS-21 scale.	Normal (0-7), Mild Anxiety (8-9), Moderate Anxiety (10-14), Severe Anxiety (15-19), Extremely Severe Anxiety (20+)<br>No Anxiety (0-7)<br>Anxiety (>7)
Stress	Level of symptoms of stress distinguished by DASS-21 scale.	Normal (0-14), Mild Stress (15-18), Moderate Stress (19-25), Severe Stress (26-33), Extremely Severe Stress (34+)<br>No Stress (0-14)<br>Stress (> 14)
Self-esteem	Rosenberg Self-esteem scale score ranges from 10-40 and is categorized as:	Low (≤ 20)<br>High (> 20)
**B. Independent Variables**		
**Socio-demographic Characteristics**		
Age	Age of adolescents completed at the time of the study	≤ 15<br>> 15
Sex	Sex of the adolescents	Male<br>Female
Ethnicity		

Type of school	Type of school where the adolescents were studying at the time of the study	Public<br>Private
Grade	Current grade of the adolescents at the time of the study	9<br>10
Family Income (NPR)	Overall income of the household	<15000<br>15000-30000<br>30000-45000<br>> 45000
**Family Characteristics**		
Parenting Style	The practices and responsibility parents exhibit towards their child rearing and caring.	Authoritarian Parenting Style<br>Authoritative Parenting Style<br>Permissive Parenting Style
Type of family	Type of family based on composition of family members	Nuclear family<br>Joint/Extended family
Mother's education	Highest level of education attained by participant's mothers	Illiterate<br>Literate (Nonformal, Primary, Secondary, High School, Higher Level)
Father's education	Highest level of education attained by participant's fathers	Illiterate<br>Literate (Nonformal, Primary, Secondary, High School, Higher Level)
Father's age	Age of fathers completed at the time of the study	≤ 40<br>> 40
Mother's age	Age of mothers completed at the time of the study	≤ 45<br>> 45
Marital status	Marital status of the participant's parents at the time of the study.	Married<br>Separated<br>Divorced
**Contextual Characteristics**		
Relationship status	Romantic relationship with another person in the past 12 months	Single<br>Dating
Relationship with friends	Social relationship status with your friends in the past 12 months	Not close<br>Close

Relationship with teachers	Social relationship status with your teachers in the past 12 months	Not close<br>Close
Involvement in Extra Curriculum Activities (ECA)	Involvement in ECA in the past 12 months	No<br>Yes
Bullying	Bullying status in the past 12 months	No<br>Yes
