# Supplementary material for: Relationship of parenting styles on depression, anxiety, stress and self-esteem of adolescents
Source: PLoS One. 2025 Dec 3;20(12):e0332854. doi: 10.1371/journal.pone.0332854 (PMC12674523; doi:10.1371/journal.pone.0332854)
Supplement: S1 Tool — English Questionnaire. https://doi.org/10.6084/m9.figshare.30490790 (PDF) [file pone.0332854.s002.pdf]

STUDY TOOL: ENGLISH QUESTIONNAIRE

**सामाजिक-जनसांख्यिकीय जानकारी (Socio-demographic Characteristics)**			
Questions	Response		
उमेर (Age)			
लिङ्ग (Sex)	1. पुरुष (Male)	2. महिला (Female)<br>3. भन्न रुचाउन (Prefer not say)	
जातियता (Ethnicity)	1. ब्राह्मण/क्षेत्री (Brahmin/Chettri)<br>2. Janajati (जनजाति)	3. दलित (Dalit)<br>4. भन्न रुचाउन (Prefer not say)	
विद्यालयको प्रकार (Type of School)	1. सामुदायिक (Public)	2. संस्थागत (Private)	
कक्षा (Grade)	1. ९	2. १०	
बसोबास स्थल (Place of residence)			
वडा नं. (Ward no.)			
बुवाको उमेर (Father's age)		आमाको उमेर (Mother's age)	
आमाको शैक्षिक योग्यता (Mother's education)	1. निरक्षर (Illiterate)<br>2. साक्षर (Literate)		
If literate, then which level? यदी साक्षर छ भने, कती सम्म पढ्नु भएको छ?	1. Nonformal अनौपचारिक<br>2. Primary level (up to 8) प्राथमिक स्तर (8 सम्म)<br>3. Secondary level (up to SEE) माध्यमिक तह (SEE सम्म)	4. High School level (up to 12) उच्च विद्यालय स्तर (१२ सम्म)<br>5. Higher Education (Bachelors and above) उच्च शिक्षा (स्नातक र माथि)	
बुवाको शैक्षिक योग्यता (Father's education)	1. साक्षर (Literate)<br>2. निरक्षर (Illiterate)		
If literate, then which level? यदी साक्षर छ भने, कती सम्म पढ्नु भएको छ?	1. Nonformal अनौपचारिक<br>2. Primary level (up to 8) प्राथमिक स्तर (8 सम्म)<br>3. Secondary level (up to SEE) माध्यमिक तह (SEE सम्म)	4. High School level (up to 12) उच्च विद्यालय स्तर (१२ सम्म)<br>5. Higher Education (Bachelors and above) उच्च शिक्षा (स्नातक र माथि)	
आमाको पेशा (Mother's occupation)	1. गृहिणी (Homemaker)<br>2. Service sector सेवा क्षेत्र<br>3. Business व्यापार	4. Farming खेती<br>5. Animal husbandry पशुपालन<br>6. Others (Specify)……………….. अन्य	

बुवाको पेशा (Father's occupation)	1. Service sector सेवा क्षेत्र<br>2. Business व्यापार<br>3. Farming खेती	4. Animal husbandry पशुपालन<br>5. Unemployed बेरोजगार<br>6. Others (Specify)……………….. अन्य
पारिवारिक आम्दानी (Family income)		
आमाबाबुको वैवाहिक स्थिति (Marital status of parents)	1. Married<br>2. Separated	3. Divorced
Family members परिवारका सदस्यहरु		
परिवारको प्रकार (Family Type)	1. Nuclear एकल<br>2. Joint संयुक्त	3. Extended बृहत
How many siblings do you have?	1. 1<br>2. 2	3. 3<br>4. 4 or more

Parenting style

नोट: अभिभावक शैली भनेको भावनात्मक र सहयोगी वातावरण प्रदान गर्दै आफ्ना बालबालिकाको पालनपोषण र हेरचाहप्रति अभिभावकहरूले प्रदर्शन गर्ने अभ्यास र जिम्मेवारी हो।

Note: Parenting style is the practices and responsibility parents exhibit towards their child rearing and caring, providing an emotional and supportive environment.

कृपया तलका प्रश्नहरू राम्ररी पढ्नुहोस् र तपाईंलाई लागू हुने बक्सहरूमा टिक गर्नुहोस्;

Please read the following questions properly and tick the boxes that apply to you;

1 = Never कहिले पनि नाइ

2 = Once in a while केहि समय मा एक पटक

3 = About half of the time लगभग आधा समय

4 = Very often धेरै पटक

5 = Always सधैं

	Never	Once in a while	About half of the time	Very often	Always
मेरा आमाबाबुले मलाई मेरो समस्याहरूको बारेमा कुरा गर्न प्रोत्साहन दिनुहुन्छ।<br>My parents encourage me to talk about my troubles.					
मेरा आमाबाबु मेरो भावना वा आवश्यकताहरूप्रति उत्तरदायी हुनुहुन्छ।<br>My parents are responsive to my feelings or needs					
म दुखी हुँदा मेरा आमाबाबुले मलाई सान्त्वना र समझ दिनुहुन्छ।					

My parents give me comfort and understanding when I am upset.					
म राम्रो हुँदा मेरो आमाबुबाले मलाई प्रशंसा गर्नुहुन्छ।<br><br>My parents give me praise when I am good.					
मेरा आमाबाबुले मसँग न्यानो र घनिष्ठ समय बिताउनुहुन्छ।<br><br>My parents have warm and intimate times together with me.					
मेरा आमाबाबुले मलाई नियमहरू पालन गर्नुपर्ने कारणहरू दिनुहुन्छ।<br><br>My parents give me reasons why rules should be obeyed.					
मेरा आमाबाबुले मलाई मेरो आफ्नै कार्यहरूको परिणामहरूको बारेमा कुरा गर्न प्रोत्साहन दिएर व्यवहारको प्रभाव बुझ्न मद्दत गर्नुहुन्छ।<br><br>My parents help me to understand the impact of behavior by encouraging me to talk about the consequences of my own actions.					
मेरा आमाबाबुले मेरो व्यवहारको नतिजाहरू व्याख्या गर्नुहुन्छ।<br><br>My parents explain the consequences of my behavior.					
मेरा आमाबाबुले नियमहरूको कारणहरूमा जोड दिनुहुन्छ।<br><br>My parents emphasize on the reasons for rules.					
मेरा आमाबाबुले मेरो असल र नराम्रो व्यवहारको बारेमा उनीहरूलाई कस्तो लाग्छ भनेर मलाई व्याख्या गर्नुहुन्छ।<br><br>My parents explain to me about how they feel about my good and bad behavior.					
मेरा आमाबाबुले मलाई व्यक्त गर्न प्रोत्साहन दिएर मेरो विचारलाई आदर देखाउनुहुन्छ।<br><br>My parents show respect for my opinions by encouraging me to express.					
मेरा आमाबाबुले मलाई आमाबाबुसँग असहमत हुँदा पनि स्वतन्त्र रूपमा (आफैलाई) व्यक्त गर्न प्रोत्साहन दिनुहुन्छ।<br><br>My parents encourage me to freely express (myself) even when disagreeing with parents.					
मेरा आमाबाबुले मलाई पारिवारिक नियमहरूमा इनपुट दिन अनुमति दिनुहुन्छ।<br><br>My parents allow me to give input into family rules.					
मेरो आमाबुबाले मलाई केहि गर्न अघि मेरो इच्छालाई ध्यानमा राख्नुहुन्छ।					

My parents take my desires into account before asking me to do something.					
मेरा आमाबाबुले परिवारको लागि योजना बनाउनमा मेरो प्राथमिकतालाई ध्यानमा राख्नुहुन्छ।<br><br>My parents take into account my preferences in making plans for the family.					
मेरा आमाबाबुले मलाई अनुशासन गर्ने तरिकाको रूपमा शारीरिक सजाय प्रयोग गर्नुहुन्छ।<br><br>My parents use physical punishment as a way of disciplining me.					
म अनाज्ञाकारी हुँदा मेरा आमाबाबुले पिट्नुहुन्छ।<br><br>My parents spank when I am disobedient.					
मैले दुव्र्यवहार गर्दा मेरा आमाबुबाले मलाई थप्पड हान्नहुन्छ।<br><br>My parents slap me when I misbehave.					
म अनाज्ञाकारी हुँदा मेरा आमाबाबुले मलाई समात्नुहुन्छ।<br><br>My parents grab me when I am being disobedient.					
मेरा आमाबाबु मप्रति रिस देखाउनुहुन्छ।<br><br>My parents explode in anger towards me.					
मैले दुव्र्यवहार गर्दा मेरा आमाबुबा चिच्याउनुहुन्छ वा कराउनुहुन्छ।<br><br>My parents yell or shout when I misbehave.					
मेरा आमाबुबाले मलाई सुधार गर्न गाली र आलोचना गर्नुहुन्छ।<br><br>My parents scold and criticize to make me improve.					
मेरो व्यवहारले उनीहरूको अपेक्षा पूरा गर्दैन भने मेरा आमाबाबुले गाली र आलोचना गर्नुहुन्छ ।<br><br>My parents scold and criticize when my behavior doesn’t meet their expectations.					
मेरा आमाबाबुले कुनै पनि स्पष्टीकरण नदिएर मबाट विशेषाधिकारहरू खोसेर सजाय दिनुहुन्छ।<br><br>My parents punish by taking privileges away from me with little if any explanations.					
मेरा आमाबाबुले थोरै वा कुनै औचित्य बिना सजायको रूपमा धम्कीहरू प्रयोग गर्नुहुन्छ ।					

My parents use threats as punishment with little or no justification.					
मेरा आमाबाबुले मलाई कुनै स्पष्टीकरण नदिएर कतै एक्लै छोडेर सजाय दिनुहुन्छ।<br><br>My parents punish by putting me off somewhere alone with little if any explanations.					
जब मैले मेरा आमाबाबुलाई सोध्छु किन मैले अनुरूप गर्नुपर्छ, भन्छन्: किनभने मैले त्यसो भनेको थिएँ, वा म तिम्रो अभिभावक हुँ ।<br><br>When I ask my parents why I have to conform, states: they say because I said so, or I am your parent and I want you to.					
मेरा आमाबाबुले मलाई सजाय दिनुहुन्छ र वास्तवमा त्यसो गर्नुहुन्न।<br><br>My parents state punishments to me and do not actually do them.					
मेरा आमाबाबुले मलाई वास्तवमा दिनु भन्दा धेरै पटक सजायको धम्की दिनुहुन्छ।<br><br>My parents threaten me with punishment more often than actually giving it.					
जब मैले कुनै कुरालाई लिएर हंगामा मच्चाउँछु तब मेरा आमाबाबुले मलाई सम्झाउनुहुन्छ।<br><br>My parents give into me when I cause a commotion about something.					
मेरा आमाबाबुलाई मलाई अनुशासन दिन गाह्रो लाग्छ।<br><br>My parents find it difficult to discipline me.					
मेरा आमाबाबुले मलाई बिगार्नुहुन्छ ।<br><br>My parents spoil me.					

उदासीनता, चिन्ता र तनाव स्केल (Depression, Anxiety and Stress Scale)

कृपाया तल दिएको बयान ध्यानपुरवक पढनुहोला । पछिल्लो एक हप्तमा यी कुराहरु तपाइको जीवनमा कुनै हड सम्म लागु हुन्छ तल दिएको खाली स्थान मध्य जुन चाहि बढि उपयुक्त हुन्छ, त्यसमा चिन्हा लगाउनु होला । यो प्रसन्नको कुनै पनी सहि र गलत उत्तर छैन। यो केवल तपाइको पछीलो अनुभव जनाउनको लागी मात्र हो।

Please read each statement and circle a number 0, 1, 2 or 3 which indicates how much the statement applied to you over the past week. There are no right or wrong answers.

०- कहिलयै पनी भएको छैन (Did not apply to me at all)

१- कुनै हद सम्म भएको छ (Applied to me to some degree, or some of the time)

२- अली बढि भएको छ (Applied to me to a considerable degree or a good part of time)

३ एकदम बढि भएको छ (Applied to me very much or most of the time)

	Did not apply to me at all	Applied to me to some degree, or some of the time	Applied to me to a considerable degree or a good part of time	Applied to me very much or most of the time
I found it hard to wind down (आफूले आफैलाई सहजता महशुस गर्न गाह्रो भएको थियो)				
I was aware of dryness of my mouth (मलाई मेरो मुख सुक्खा भएको अवगत हुन्थयो)				
I couldn't seem to experience any positive feeling at all<br><br>मैले केहि सकारात्मक भाव अनुभव गर्न सकेको थिएन				
I experienced breathing difficulty (e.g., excessively rapid breathing, breathlessness in the absence of physical exertion)<br><br>मलाई श्वास फेर्न गाह्रो महसुस हुन्थ्यो, जस्तै छिटो छिटो फेर्न, कुनै शारीरिक परिश्रम बिना श्वास फुल्ने				
I found it difficult to work up the initiative to do things<br><br>मलाई कुनै पनि काम सुरुवात गर्न गाह्रो भएको थियो				
I tended to over-react to situations<br><br>मैले चहिने भन्दा बढी प्रतिक्रिया दिन्थे				
I experienced trembling (e.g. in the hands)<br><br>मैले मेरो शारीरमा कम्पन महसुस गरेको थिए (जस्तै हात कप्ने)				
I felt that I was using a lot of nervous energy				

मैले यस्तो महसुश गरे कि, म कुनै बेल केहि बढी नै डराए				
I was worried about situations in which I might panic and make a fool of myself<br><br>म यो परिस्थितिको बारेमा बढी नै चिन्तित हुन्थे, जसले गर्दा म धेरै डराएर आफुले आफैलाई मुर्ख बनाउने त हैन भन्ने हुन्थ्यो				
I felt that I had nothing to look forward to<br><br>मलाई अघि बढने आधर नै छैन भन्ने अनुभव हुन्थ्यो				
I found myself getting agitated<br><br>म आफै सानो कुराहरुमा चिन्तित भएको पाए				
I found it difficult to relax<br><br>मलाई आराम गर्न गाह्रो भएको थियो				
I felt down-hearted and blue<br><br>मैले आफु उदाशिन र दुखी महसुस गरेको थिए				
I was intolerant of anything that kept me from getting on with what I was doing<br><br>मैले गरेको काममा कसैले कुनै तरिकबाट बाधा दिएम, मलाई सहन नसक्ने हुन्थ्यो				
I felt I was close to panic<br><br>मैले म आतंकित भएको महसुस गरेको थिए				
I was unable to become enthusiastic about anything<br><br>म कुनै पनि काम गर्न उत्साहित थिएन				

I felt I wasn't worth much as a person<br><br>म अफु योग्य व्यक्ति नभएको महसुस भयो				
I felt that I was rather touchy<br><br>म बढीनै भाबुक भएको महासुस भयो				
I was aware of the action of my heart in the absence of physical exertion (e.g. sense of heart rate increase, heart missing a beat)<br><br>शारीरिक कृयाकलाप विना नै मैले मेरो मुटुको धड्कन महासुस गरेको थिए- जस्तै छिटो छिटो धड्केको वा कहिले काहि धड्कन रोकिएको				
I felt scared without any good reason<br><br>मैले कुनै कारण बिना नै डर को महासुस गरेको थियो				
I felt that life was meaningless<br><br>मैले मेरो जिवनको सार्थकतानै छैन भन्ने अनुभव भएको थियो				

What is your relationship status?<br>तपाईको सम्बन्धको स्थिति के छ?	1. Single एकल<br>2. Dating सम्बन्ध	
How is your relationship with your friends?<br>तपाईका साथीहरूसँगको सम्बन्ध कस्तो छ?		
Are you involved in extra-curriculum activities?<br>के तपाइँ अतिरिक्त क्रियाकलापमा संलग्न हुनुहुन्छ?	1. छु<br>2. छैन	
If yes, what kind of?<br>यदि हो भने, के मा?	1. Sports खेलकुद<br>2. Community engagement समुदाय संलग्नता	3. Social works सामाजिक कार्यहरू<br>4. Others………….. अन्य

Have you face any kind of bullying in school or outside school?<br>के तपाईंले विद्यालयमा वा विद्यालय बाहिर कुनै प्रकारको दुर्व्यवहारको सामना गर्नुभएको छ?	1. छु<br>2. छैन
If yes, then where?<br>यदि हो भने, कहाँ?	1. School विद्यालय<br>2. Cyber अनलाइन<br>3. Community समुदाय<br>4. Other settings………..<br>अन्य

आत्मसम्मान (Self-esteem)

नोट: आत्म-सम्मान भनेको आफैको समग्र मूल्याङ्कन हो, अर्थात्, आफ्नो बारेमा सकारात्मक, नकारात्मक र मिश्रित विचार वा भावनाहरू, र यदि कसैसँग बढी सकारात्मक भावनाहरू छन् वा आफूलाई योग्य ठान्छन् भने, आत्म-सम्मान उच्च हुनेछ।

Note: Self-esteem is the subjective appraisal of oneself, i.e., positive, negative and mixed thoughts or feelings about one's own self, and if one has more positive feelings or considers oneself as worthy, self-esteem will be higher.

तल तपाइँको बारेमा तपाइँको सामान्य भावनाहरु संग व्यवहार गर्ने बयान को एक सूची छ। तपाईं प्रत्येक कथनसँग कत्तिको सहमत वा असहमत हुनुहुन्छ भनेर कृपया संकेत गर्नुहोस्।

Below is a list of statements dealing with your general feelings about yourself. Please indicate how strongly you agree or disagree with each statement.

	पूर्ण सहमत (Strongly Agree)	सहमत (Agree)	असहमत (Disagree)	पूर्ण असहमत (Strongly Disagree)
समग्रमा म आफैंमा सन्तुष्ट छु ।<br><br>On the whole, I am satisfied with myself.				
कहिलेकाहीँ मलाई लाग्छ कि म राम्रो छैन।<br><br>At times I think I am no good at all.				
मलाई लाग्छ कि ममा धेरै राम्रा गुणहरू छन्।<br><br>I feel that I have a number of good qualities.				
म पनि धेरैजसो अन्य मानिसहरू जस्तै चीजहरू गर्न सक्षम छु।<br><br>I am able to do things as well as most other people.				
मलाई लाग्छ मसँग गर्व गर्न धेरै कुराहरू छैन।				

I feel I do not have much to be proud of.				
म कहिलेकाहीं बेकार महसुस गर्छु।<br><br>I certainly feel useless at times.				
मलाई लाग्छ कि म मूल्यवान व्यक्ति हुँ, कम्तिमा पनि अरूसँग बराबरको स्तरमा।<br><br>I feel that I'm a person of worth, at least on an equal plane with others.				
म चाहन्छु कि म आफैंलाई थप सम्मान गर्न साकु।<br><br>I wish I could have more respect for myself.				
सम्पुर्णमा, म असफल छु भन्ने महसुस गर्छु।<br><br>All in all, I am inclined to feel that I am a failure.				
म आफूप्रति सकारात्मक दृष्टिकोण राख्छु।<br><br>I take a positive attitude toward myself.				
