# Supplementary material for: Relationship of parenting styles on depression, anxiety, stress and self-esteem of adolescents
Source: PLoS One. 2025 Dec 3;20(12):e0332854. doi: 10.1371/journal.pone.0332854 (PMC12674523; doi:10.1371/journal.pone.0332854)
Supplement: Attachment — Submitted filename: Response to Reviewers.docx [file pone.0332854.s006.docx]

Dear Reviewers,

We sincerely appreciate your valuable feedback. We’ve done our best to address your comments and have provided the responses on the table below. The major revisions can be found in the file named “Revised Manuscript with Track Changes”.

| **S.N.** | **Reviewer Comment** | **Author Response** |
| --- | --- | --- |
|  | **Reviewer 1** |  |
| **1.** | **Title** |  |
|  | The title of the article is: “Influence of parenting styles on depression, anxiety, stress, and self-esteem of adolescents.”  Comment: I suggest changing the word “influence,” as cross-sectional studies cannot claim "influence"; this is typically associated with experimental or longitudinal studies. Using this term may confuse readers. | Thank you for your valuable feedback regarding the use of the word “influence” in the study title. We agree with your observation that the term implies a causal relationship, which is inappropriate for a cross-sectional design. In response, we have revised the title to better reflect the nature of our study and avoid any suggestion of causality.  Revised Title:  “*Relationship of parenting styles on depression, anxiety, stress, and self-esteem of adolescents”* |
| 2. | Introduction |  |
|  | The introduction needs to maintain a logical argument and structure that is easy to follow for the reader. For example, in line 47, risk factors are introduced, only to be mentioned again in line 57. The same happens with mental health issues: they are introduced in line 49 and end in line 53, only to reappear in line 58, and again, parenting styles are introduced in line 55. Further, mental health issues are mentioned again between lines 58 and 65, and parenting styles are reintroduced in line 65.  Comment: The introduction could be structured more effectively to avoid repetition and improve readability. It should begin by clearly defining the population and the issue (e.g., "Adolescence is…"), then introduce the dependent variables (DAS, self-esteem), and finally, explain the independent variable as an explanatory framework for the dependent variables. In line 76, the limited research on this topic is mentioned, but it would be helpful to add a brief paragraph discussing the limitations of prior research and how this study addresses that gap through its objectives. | Thank you for your thoughtful feedback on the structure of the introduction. In response to your suggestion, we have revised the section to enhance clarity and readability by following a more logical flow. Restructured the introduction section in clearer progression as:   - We began by clearly defining the population (adolescents) and highlighting the mental health issue in the context of global and local statistics. - We then introduced the dependent variables—depression, anxiety, stress, and self-esteem—by emphasizing their interrelated nature and role in adolescent mental health. - Following this, we positioned parenting style as the independent variable, discussing its potential impact on the psychological well-being of adolescents based on existing literature.   We also consolidated the redundant discussions of risk factors, mental health issues, and parenting styles to avoid repetition. For example: merged lines 47 and 57; merged lines 49 and 58. We rephrased the introduction section and added strong transition phrases to ensure logical flow and readability. For example: “*Given the parenting style’s significant yet understudied role*…..” in line 111; “*These stressors may lead to…*” in line 52.  We added a brief paragraph noting the limited research on the relationship between parenting style and psychological factors among adolescents, addressed the gap in the literature through the Nepal context, and highlighted the need to address this gap through the study. These updates can be found on lines 111-123. |
| 4. | Method |  |
|  | In line 106, what are the inclusion criteria? | Thank you for your feedback. As per the suggestion, the inclusion criteria have been clearly outlined on lines 150-153 of the revised manuscript to provide better transparency and clarity. |
|  | In the section on Data collection tools (line 127), it is unclear how self-esteem is coded. Are there reverse-scored items? Is it summed or subtracted? | Thank you for your insightful comment regarding the self-esteem scale. To clarify, the Rosenberg Self-Esteem Scale includes reverse-scored items, and the scoring procedure is as follows:   - The scale uses a 4-point Likert-type scale, where responses range from *strongly agree* (score: 4) to *strongly disagree* (score: 1). - Negative items (2, 5, 6, 8, and 9) are reverse scored. - The total score is the sum of all 10 items, with scores ranging from 10 to 40.   Based on the total score, the following classification is applied:   - **Scores >20** are characterized as *high self-esteem*. - **Scores <20** are categorized as *low self-esteem*.   This scoring method has been explicitly updated in lines 193-196 of the revised manuscript to address any confusion. |
|  | Was unidimensionality checked? | Yes, unidimensionality of the tools was checked. We used pre-validated tools for the study. To verify this in the context of Nepal, we translated the tools and pretested them in 58 school-going adolescents. Results presented Cronbach’s alpha for PSDQ, DASS-21, and Rosenberg Self-esteem scales as 0.85, 0.90, and 0.63, respectively. This information is updated in lines 212-213 in the revised manuscript. |
|  | In Data analysis, it would be important to detail how missing data, outliers, and errors were handled. Was the internal consistency of the instruments verified? | Thank you for your insightful comment. We have now revised the “Data Analysis” section to clarify how data quality issues were addressed.  Specifically, missing data were handled using listwise deletion of the entries with more than 20% missing values. Even after deletion, there was sufficient sample size, maintaining a lower impact of missing values.  Data were entered into IBM SPSS Statistics version 16 using a pre-designed entry form. To minimize data entry errors, a double-entry verification process was conducted, where two separate entries were compared and discrepancies resolved by referring to the original questionnaires. This procedure helped ensure accuracy and consistency in the dataset.  Furthermore, we assessed the internal consistency of all psychometric tools used in the study. The Cronbach’s alpha values were as follows:  DASS-21: α = 0.90  Rosenberg Self-Esteem Scale: α = 0.63  Parenting Style and Dimensions Questionnaire – Short Version: α = 0.85. This information is now updated in the revised manuscript and can be found in lines 210-218. |
|  | It would also be helpful to specify how covariates were selected. Was any preliminary testing (such as correlation analysis or analysis of potential confounding variables) done before selecting the variables for univariate analysis? | Thank you for your comment. In our analysis, we selected covariates for univariate analysis based on their theoretical relevance to the outcomes of interest (depression, anxiety, stress, and self-esteem) and findings from previous literature. While we did not conduct formal correlation analysis or detailed preliminary testing for potential confounding variables, we included covariates such as socio-demographic factors (e.g., age, gender, socioeconomic status) and family characteristics, which are commonly recognized as potential influencers of adolescent mental health outcomes.  This information is now included under the Data Analysis section as “These covariates were based on the theoretical relevance…” In lines 226-228. |
|  | In the multivariable logistic regression analysis, was multicollinearity assessed? | Thank you for your comment. We assessed multicollinearity for independent variables like age, grade, parents’ education, and other potential predictors. We used the variance inflation factor (VIF) and tolerance values to assess the presence of multicollinearity. The results indicated that all VIF values were less than 5 and tolerance values were above 0.2, suggesting no significant multicollinearity in the model. However, our manuscript lacked this information, and it is now mentioned in the data analysis section of the revised manuscript as “*Multicollinearity for independent variables like age, grade, parents’ education, and other potential predictors was assessed through the variance inflation factor (VIF) and tolerance values*, …” on lines 236-239. |
| 5. | Results |  |
|  | It is recommended to report the results directly in percentage terms rather than saying “two-thirds” or “five-sixths.” | Thank you for your valuable feedback. Following your suggestion, we have revised the presentation of the results to report percentages directly rather than using terms like “two-thirds” or “five-sixths”. For example, on line 238, “about two-thirds” is changed into “about forty-two percent”, and “five-sixths” is changed into “eighty-three percent” in line 240; similar changes have been made for other variables throughout the manuscript to provide clearer and more precise results. The update can be found on lines 259 to 275. |
|  | In line 177, the range 15,000-30,000 is mentioned. It would be helpful to include the equivalent in USD, as readers are not focused solely on Nepal. | Thank you for your suggestion. In response, the income range previously stated in the manuscript as Nepali rupees has now been converted to USD using an exchange rate of 1 Nepali rupee valued at 0.0072 USD. This change is made in line 265 and the income category row in Table 1. |
|  | In line 198, it is stated that the scale consists of three subscales with their dimensions; it would be useful to reference this scale earlier, such as in line 119. | Thank you for your valuable feedback. Based on the recommendation, the subscales and dimensions of the parenting styles are now referenced earlier in lines 169 to 174 of the “Data Collection Tools” section to provide better clarity and context for the readers. We have mentioned it as “PSDQ consists of three subscales... Each subscale consists of dimensions: authoritative parenting style consists of connection, regulation, and autonomy dimensions; …” |
|  | In Table 3, a categorization of depression, anxiety, and stress levels is mentioned, but it is not addressed in the data analysis. Which authors support this categorization, and are there any established cutoffs in Nepal? | Thank you for your insightful comment. The categorization of depression, anxiety, and stress levels in Table 3 is based on the standardized cut-offs developed and validated by Lovibond and Lovibond (1995) in their DASS-21 scale.[1] We have now clarified this in the “Data Collection Tools” section of the revised manuscript in line 188-189 by referencing the appropriate source.  Regarding Nepal-specific cut-offs, to the best of our knowledge, there are no established specific cut-offs mentioned for DAS in the Nepali context. Therefore, we relied on the internationally accepted cutoffs as proposed by Lovibond and Lovibond for our analysis. |
|  | Similarly, with self-esteem, the cutoff points for high and low self-esteem are not mentioned. This should be addressed in the data analysis. | Thank you for your comment. The cut-offs for high and low self-esteem are now mentioned in the “Data Collection Tools” section. Regarding Rosenberg's self-esteem scale, the original scale doesn’t provide cut-offs for categorizing self-esteem scores; so, we have adopted the widely accepted threshold based on the previously validated and available literature. Based on these thresholds, we have coded our responses from 1 to 4; the total self-esteem scores ranged from 10 to 40, with scores above 20 considered indicative of high self-esteem, while scores below 20 indicate low self-esteem. This is mentioned in the “Data Collection Tools” section on lines 193-196 of the revised manuscript. |
|  | In Table 4 (line 229), global scores are calculated. It would be beneficial to elaborate on this when referencing the instruments used. | Thank you for your comment. Table 4 highlights the association between parenting style and outcome variables. We have now clarified these calculations in “Data Collection Tools” and “Data Analysis” section of revised manuscript, highlighting the standard tools used and categorization of data, and analysis plan followed. |
| 6. | Discussion |  |
|  | In general, the discussion is well-developed, but it needs better structure, as it currently lacks a clear logical flow. As a reader, it is easy to get lost in the content. I would recommend organizing it around the study's objectives. The objectives are: "This study aimed to assess depression, anxiety, stress, and self-esteem in adolescents and their relationship with parenting style in Bheemdatt Municipality, Kanchanpur, Nepal." | We sincerely appreciate your constructive feedback. In response, we restructured the “*Discussion”* section to follow a clear, objective-driven flow: beginning with the study’s objective, presenting the prevalence of the psychological outcomes among adolescents, explaining associations with parenting styles, discussing external factors, and concluding with limitations. |
| 7. | Conclusions |  |
|  | In this sentence: "Hence, it is concluded that parents play an influential role in promoting the mental health of their adolescent children with the supporting role of study covariates and social support systems."  Comment: Care should be taken with the language used, as "influence" cannot be concluded here; rather, "association" or "relationship" should be used. Also, referring to "mental health" is ambiguous, as it is often understood in a bipolar model (presence and absence). This study actually addresses psychological distress and self-esteem, which are well-supported topics in the literature. | Thank you for this insightful comment. We agree that the language in the conclusion should reflect the observational nature of our study. Accordingly, we have revised the sentence for clarity and accuracy. The term "influential role" has been replaced with "significantly associated," and "mental health" has been specified as "psychological distress and self-esteem" to better reflect the constructs measured in our study. The revised sentence now reads:  *“Hence, it is concluded that parenting styles are significantly associated with adolescents’ psychological distress and self-esteem, with the supporting role of study covariates and social support systems.”* |
| 8. | References |  |
|  | Comment: In this section, references should be adjusted to meet Vancouver style guidelines (e.g., World Health Organization. Adolescent health. Available: https://www.who.int/health-topics/adolescent-health#tab=tab_1) for various types of sources: books, book chapters, original articles, and reports. To facilitate this, I recommend using a reference manager such as Mendeley and downloading the Vancouver style from Plos One. | Thank you for pointing this out. We faced some issues with referencing software. We have now revised all references in the manuscript to comply with Vancouver style guidelines. To ensure consistency and accuracy, we utilized Zotero as our reference manager and applied the Vancouver citation style available through PLOS One. We have formatted all citations- including journal articles, books, reports, and web-based sources-accordingly throughout the manuscript and reference list. |
| 9. | Supplementary Files |  |
|  | It is necessary to mention the supplementary files in the text. For example, when discussing the instruments, it should be noted that more details can be found in Table A of the supplementary files. Additionally, it is important to cite and reference the authors used for the cutoff scores. | Thank you for your helpful suggestion. We have now included in-text references to the supplementary files at appropriate points in the manuscript. For instance, while discussing the instruments, we have added: “(see Table A in Supplementary File 1 for more details).” Furthermore, we have cited and referenced the authors whose work informed the cutoff scores for the scales used in this study. |
|  | **Reviewer 2** |  |
| **1.** | **Introduction** |  |
|  | The justification of the study is not very strong, especially when there are literature review and studies already conducted on the topic. How will this study make a difference and contributions? | Yes, there are studies conducted on the topic, but in different population and cultural settings. There is the study conducted by Banstola et al. in Nepal in the specific study setting, however, this result cannot be generalized to the entire population and barely identifies the association of psychological factors and parenting style. We focused on the specific region of the far-west part of Nepal where the issues of mental health and adolescents are barely addressed and lack the evidence to support such programs.  This update can be found on lines 111-123 as: *“Given parenting style’s significant yet understudied role in adolescent mental health, this factor needs to be explored more, and limited research explaining the correlation between parenting and the above-discussed psychological factors remains a big question to be answered.[12]…”* |
| 2. | Methods |  |
|  | What was the reasoning behind excluding schools with less than 50 children? What were the criteria of selecting the 10 schools out of the 124 schools? | Excluding schools with less than 50 children is to fulfill the required sample size of the research through proportionate sampling. Time and feasibility might also be reasons to select 10 schools out of 152-153. |
|  | What is the meaning of high scores in DASS, PSDQ, and Rosenberg Self-esteem Scale? What are the cut-off scores to define different levels of MH in Table 3 , 4, and 5? | The high scores of the DASS mean extremely severe depression, stress, or anxiety, and self-esteem is the high level of self-esteem. While there is no high score for PSQD, each subscale has mean scores and divided into dimensions. The mean score of the respective parenting styles is then divided by the overall mean and converted to a percentage. These are mentioned in the data collection tools. The cut-offs for mental health levels are also mentioned in the data collection tools, which are based on internationally accepted standards. These updates can be found on lines 174-196 of the revised manuscript. |
|  | Were all students attending the 10 schools participated in the survey? If not, what was the participation number/rate? | No, all the students from 10 schools didn’t participate in the survey. After the selection of the schools, the sections of grades 9 and 10 were randomly selected. Likewise, those students with parental consent participated in the study. Remembering the participation rate, everyone from the selected sections participated in the survey. |
| 3. | Data Analysis |  |
|  | What were the covariates you tested and used in the multivariate logistic? | Thank you for your comment. The covariates tested and used in the multivariate logistic regression were age, sex, ethnicity, type of school, grade, mother’s and father’s education, mother’s and father’s occupation, mother’s and father’s age, income, parent’s marital status, type of family, siblings, relationship status, relationship with friends and teachers, involvement in extra-curriculum activity, and bullying. This update is included on lines 222-225 of the revised manuscript. |
|  | Why did you not use multilevel model when your data are clearly clustered? Did you measure the ICC? | We thank the reviewer for highlighting this point. Post hoc ICC calculation explained that there was negligible correlation among clustered data. While we acknowledge that MLM is often ideal for clustered data, our findings suggest that school type had little practical impact on this sample. |
| 4. | Results |  |
|  | “the percentage of authoritative parenting style was 83.2%) What were the criteria to define authoritative parenting style? The same with the other two style. In addition, what is the connection between parenting style and dimensions in Table 2? More explanations are needed for readers who are not familiar with the topic. | We thank the reviewer for raising these important points. We used the *Parenting Styles and Dimension Questionnaire (PSDQ)* by Robinson et al., where parenting styles are based on the mean scores. Participants’ mean scores for authoritative, authoritarian, and permissive parenting styles were calculated separately. A parent was classified authoritative if their mean score for authoritative score was higher than their mean scores for both authoritarian and permissive scales. This is consistent with PSDQ’s standard scoring approach. Similarly, the 83.2% prevalence reflects the proportion of parents in our sample whose authoritative subscale mean score dominated over the other two styles.  The connection between parenting style and dimensions in Table 2 is presented as mentioned by the original author of the scale. Table 2 displays mean scores for individual dimensions (e.g. warmth, discipline) that comprise each parenting style subscale. For example: the authoritative parenting style’s high overall mean resulted from the high scores on the dimensions like warmth and reasoning, as shown in Table 2.  We have revised the manuscript to give readers a clear view of the methods. These updates can be found on lines 174-183 of the revised manuscript. |
|  | Association between parenting style and outcome variables: analysis to measure this was not mentioned in the statistical analysis section. what sort of analysis did you use? For the variables, did you use the scores or categories? | We performed a point-biserial correlation analysis to assess relationships between: Parenting style subscale mean scores (authoritative, authoritarian, permissive) and dichotomized DASS outcomes (e.g., "depression" vs. "no depression" based on standard cutoffs)  This approach was selected because the point-biserial correlation is mathematically equivalent to Pearson's (r) when one variable is continuous and the other is dichotomous, and it provides a direct measure of effect size while respecting the mixed variable types. This has been updated on lines 232-234 of the revised manuscript as:  *“Likewise, point-biserial correlation was used to assess the nature of association between independent (continuous) and dependent variables (dichotomous), and…”* |
|  | Table 5: did you use the parenting style one of the variables in the model? Your research question was: investigating the relationship between parenting style and MH. Table 5 shows the association between demographic factors and MH, which was not part of the research question. Instead, Table 5 should report results from logistic regressions using parenting style as the main predictor and the demographic factors as the covariates (control variables). | We have duly noted the suggestion. Reviewing our research question, we begin the study with idea of assessing the status of depression, anxiety, stress, and self-esteem among adolescents, and then measuring this association with parenting style. This is mentioned in the line 121-125 as *“Likewise, issues and factors of the mental health are barely discussed and identified... Therefore, this study aimed to assess depression, anxiety…”*  We feel Table 5 is an equally important aspect of study. Instead, we have added Table 6 to the results highlighting the results of logistic regression using parenting style as the main predictor and the demographic factors as the covariates. |
|  | Table 5: have you tried to run a logistic regression predicting depression (or anxiety/stress/self-esteem) using the parenting style as the main predictor and you used all of the characteristics listed in Table 5 (instead of choosing only those that were significant in bivariate analysis? | Initially, our analysis didn’t include logistic regression predicting outcome variables and parenting style as the main predictor. After careful consideration of this suggestion, we recognized the value this analysis will add to our findings. So, we have done the additional analysis and presented the results in Table 6 under the title “*Logistic Regression for Association between Parenting Styles and Outcome Variables*” from lines 347 – 363 in the revised manuscript. |
|  | Have you check the multicollinearity issue (e.g., age and grade would be highly correlated)? Why did you choose age and grade separately, instead of using one of them as predictor? | Thank you for raising this point. We assessed multicollinearity for independent variables like age, grade, and other potential predictors. We used the variance inflation factor (VIF) and tolerance values to assess the presence of multicollinearity. The results indicated that all VIF values were less than 5 and tolerance values were above 0.2, suggesting no significant multicollinearity in the model.  We choose age and grade separately as they reflect different developmental states. Age reflects the biological maturity, while grade reflects educational experience. We choose them separately due to their different contributions to the outcome, and the overall pattern of results remains consistent.  This update can be found in the data analysis section of the revised manuscript as “*We choose age and grade as separate entities because of their different developmental nature and impact to the outcome of the study. Multicollinearity for independent variables like age, grade…*” on lines 234-239. |
| 4. | Discussion |  |
|  | There are close to 2 pages of discussion about the prevalence of depression, anxiety, stress, and self-esteem (including comparisons with different countries) – page 18 to 20. Although this is useful, I feel that the discussion could be made more concise., which will make the paragraph easier to digest and the readers can get the message easily. | Thank you for your feedback. We have carefully reviewed the existing content in the discussion. We have streamlined the prevalence analysis and removed repetitive content and consolidated key comparisons in the discussion.  Updated version:  *“Past studies conducted by Sharma et al. (depression: 41.6%, anxiety: 56.9%) and Karki et al. (depression: 56.5%, anxiety: 55.6%) reported higher rates.[20,25] While Gautam et al.'s study in rural Nepal…”* |
|  | Starting from line 346 is the discussion about demographic factors influence on MH, which was not mentioned in the research question – so, why was there a bigger proportion discussing the sociodemographic factors compared to the discussion about the parenting style? | Thank you for your feedback. Actually, it was our research question mentioned on lines 123-125. We not only assess the relationship between the variables but also plan to evaluate the situation of the outcome variables. To clarify this confusion, we added more about our research questions in the introduction section. |
|  | Line 346-355, discussion about mothers' and fathers' education: they were not significant predictors of MH in Table 5 - so I would expect the discussion would be the reasons for not finding the effect, rather than sayng that the risk of depression was higher in adolescents with mothers without any formal education (when the OR was not significant) | Thank you for your feedback. We now see the difference in the discussion and have highlighted the non-significance of the variables with DAS and self-esteem. Also, we have removed the major discussion portion from the paragraph and kept it more concise.  We updated this in lines 494-504 as “*Mothers’ and fathers’ education wasn’t found to be significant with the DAS and self-esteem among adolescents. However, existing research studies by Gautam et. al.[72] , Bhattarai et. al [73] showed the significant association of depression with parental education..*.” |
|  | Line 356 – 359: only ORs for Anxiety, stress, and self-esteem that were significant – but the mention gave the impression that OR for depression was also significant. Also, the OR for depression was not 1.52 but 1.17. | Thank you for pointing this out. We have revisited our findings and corrected this error. We have also revised the text to clearly indicate which variables reached statistical significance. Updates can be found on lines 508-515 in revised manuscripts such as:  *“Relationships with friends showed a significant association with anxiety, stress, and self-esteem, and relationships with teachers showed a significant association with stress… however, depression wasn’t significant. However, some research…”* |
|  | Line 356-373: I would like to see the relationship with teachers to be discussed as well | Thank you for your feedback. We have reviewed the lines and added on the discussion of the variable “relationship with teachers”. You can find the update on lines 509-531. |
|  | Limitations: I would like to see more limitations being identified, especially in relationship to the study design and methods. | We sincerely respect the reviewer’s constructive feedback. We have expanded the limitations to address methodological and design constraints more thoroughly. You can find the updates on lines 556-563 in the revised manuscript, such as:  *“The measures used in the study relied on the adolescents’ self-reports on their mental status, which may introduce recall bias on this sensitive topic. Parental perspective on this topic …”* |
|  | What are the implications and contributions of the study? Why do we need to care about this study? | We appreciate the feedback to clarify the broader significance of our work. Our findings matter because they address Nepal’s adolescent mental health through actionable and evidence-based strategies. While most evidence on parenting styles and adolescents’ mental health outcomes comes from the Western setting, our study provides critical data from Nepal to contribute to identifying the risk factors associated with mental health. It could also help policymakers to adopt evidence-based parenting guidelines to Nepali cultural norms. Our findings also advocate for integrating mental health screening in the school health programs, particularly in underserved regions like Sudurpaschim Province. Likewise, Nepal’s adolescent suicide rates are among the highest in Southeast Asia. By identifying modifiable family-level factors like parenting style alongside systemic issues like bullying, our study provides strategic points to mitigate this problem.  We have incorporated this in the “Conclusion” section from line 586-593. |
