# Supplementary material for: Relationship of parenting styles on depression, anxiety, stress and self-esteem of adolescents
Source: PLoS One. 2025 Dec 3;20(12):e0332854. doi: 10.1371/journal.pone.0332854 (PMC12674523; doi:10.1371/journal.pone.0332854)
Supplement: Attachment — Submitted filename: Response_to_Reviewers_auresp_2.docx [file pone.0332854.s007.docx]

Dear editor/reviewer,

We sincerely appreciate your valuable feedback. We’ve addressed your comments to our best ability in the table presented below. The key revisions are highlighted in the file named “Revised Manuscript with Track Changes”.

| **S.N.** | **Comments** | **Author Response** |
| --- | --- | --- |
|  | **Journal Office** | |
| 1. | Additionally, the journal office requests that you revise lines 417 - 420:  "The possible explanation must be the difference in parenting between male and female adolescents which can result in differences in the mental status of the adolescents. Likewise, females are more likely to be exposed to the family burden and social stigma."  The last line of this is missing a reference to support it. Additionally, we would suggest rephrasing the opening to "One possible explanation could be" as there are other possible explanations for these results, which isn't clear in the current wording. | Thank you for your suggestion. We have reviewed the sentence and carefully paraphrased the sentence and cited the relevant source. The updates can be found on lines 417-419 of the revised manuscript. |
|  | **Reviewer comments** | |
|  | **Reviewer #1** | |
| 2. | Dear Editor in Chief  I sincerely appreciate the opportunity to review the manuscript titled “Relationship of parenting styles on depression, anxiety, stress, and self-esteem of adolescents”, conducted in a socially and culturally diverse context such as Nepal.  Overall, I have observed that, in the introduction, method, data analysis, results, discussion, and conclusions, the authors have substantially improved the manuscript by carefully addressing...This study presents important and relevant research...Thank you for your thorough work, and congratulations to the authors for their dedication and the quality of this research. | We are thankful to Reviewer #1 for their generous and encouraging comments. We are very happy that our previous revisions were found to be satisfactory and have strengthened the manuscript. |
|  | **Reviewer #3** | |
| 3. | Sample size, can the authors indicate, whether on average it was expected to recruit approx 58 participants from either public or private - would you expect a similar distribution or more in one type than another. Only because there is statement regarding exclusion of school that had <50 student, would this contribute to some selection bias? | We thank the reviewer for this observation. We want to clarify the final sampling distribution resulting from proportionate sampling based on the total number of students in each randomly selected school, not an equal number from each school. This indicates that public schools in the municipality have larger enrollments. For clarification, we have mentioned this on lines 123-125 of the revised manuscript. Likewise, the exclusion of schools with <50 students was mainly due to logistical feasibility to ensure we reach the desired sample size within a manageable number of clusters. This might be subject to some bias, limiting the generalizability of findings to the adolescents within such settings. We have included this in the limitations of the revised manuscript as “Furthermore, the exclusion of schools with very few enrollments may limit…” |
| 4. | For ease of reading, suggest to include in the text, what p and q is? How was the design effect obatained and what was the variance cluster | Thank you for the suggestion. We have now included the meaning of p and q in line 117 of the main manuscript. Similarly, the value for design effect (1.5) was chosen based on the commonly used estimate in many public health studies, as the actual ICC cannot be known until the data is collected. |
| 5. | The essence of univariate analyses, if the main goal is to understanfding which confounding variables go into the multivariate model, should be to assess significance. NOt sure assessing odds only in univariate is appropriate. Line 225, 226 - can this include line 229. | Thank you for this valuable comment. We have reviewed the lines and made changes accordingly. |
| 6. | Was potential mediators also looked into? i.e impact of income as a confounder on the parenting styles and adolescent mental health, or even together with influecing the type school you went to, i.e private or public?; considered as some exploratory analyses. | Thank you for this insightful comment. We did check for direct associations for potential mediators; however, we didn’t run any moderation analysis. As mentioned earlier, our objective was more focused on direct associations of factors. This opens the pathways for future research and has been mentioned within the discussion section on lines 472-473. |
| 7. | The income reported in dollars is, that monthly or annual? it would be good to clarify? Does it 72% did not have an income? or the range is outside this. It would be good to give context, is this low, medium or high earnings? | Thank you for the feedback. The reported income in dollars represents the monthly income of the family of respondents. We have mentioned this in line 240 of the revised manuscript. The remaining 72% fall into other income categories: 25.7% earn <107.28 USD, 25% earn 214.55-321.83 USD, and 21.3% earn >321.83 USD, which is presented in Table 1. Likewise, we used the income category from similar research in adolescents,[1] but there was no established standard for high, medium and low earnings. |
| 8. | Table 1: Report Range of Age ( students, mothers, fathers) in the table? | Thank you for the feedback. We have now incorporated the range for the respective age groups in Table 1 of the updated manuscript. |
| 9. | Table 1: Education level, does illiterate mean (no education?) | Thank you for the feedback. Yes, the word “illiterate” used in the manuscript refers to no formal education. |
| 10. | Table 1: include for words for ECA in table or footnote so reader is not searching for what ECA means. | Thank you for the suggestion. We have included the word in the footnote. |
| 11. | Table 2: Helps to present ranges, assuming these are normally distributed, suggested to also present median (IQR) too. | Thank you for your suggestion. We have now presented the median and inter-quartile range in Table 2 of the revised manuscript. |

We believe addressing these points has substantially strengthened our manuscript. Thank you.

Sincerely,

Anjali Bhatt

**References**

1.	Gautam L, Thapa M, Pokhrel P, Sapkota PM, Bhatt A, Paudel K. Cross-sectional study on behavioural determinants associated with sugary foods and beverages and their correlates with body mass index among adolescents in Nagarjun municipality, Nepal. 2024 [cited 15 Oct 2025]. doi:10.1136/bmjopen-2024-086755
